# Tumour Transfer to Bone Graft Donor Site: A Case Report and Review of the Literature of the Mechanism of Seeding

**DOI:** 10.1155/S1357714X00000098

**Published:** 2000-03

**Authors:** Richard G. Dias, Adesegun Abudu, Simon R. Carter, Robert J. Grimer, Roger M. Tillman

**Affiliations:** Royal Orthopaedic Hospital Oncology Service The Royal Orthopaedic Hospital NHS Trust Bristol Road South Northfield Birmingham B31 2AP UK

## Abstract

*Purpose.* Transmission of malignant tumour cells to a bone graft donor site is a rare complication of bone grafting.We report a case of seeding of malignant fibrous histiocytoma from the femur to a pelvic bone graft donor site.

*Discussion.* We review the literature, discuss the possible mechanism of tumour transfer and offer advice aimed at avoiding this complication.


Sarcoma (2000) 4, 57–59

CASE REPORT

### Tumour transfer to bone graft donor site: a case report and review of the literature of the mechanism of seeding

RICHARD G. DIAS, ADESEGUN ABUDU, SIMON R. CARTER, ROBERT J. GRIMER & ROGER M. TILLMAN

*Royal Orthopaedic Hospital Oncology Service, The Royal Orthopaedic Hospital NHS Trust, Bristol Road South, Northfield, Birmingham B31 2AP, UK*

**Abstract**
*Purpose*. Transmission of malignant tumour cells to a bone graft donor site is a rare complication of bone grafting. We report a case of seeding of malignant fibrous histiocytoma from the femur to a pelvic bone graft donor site.
*Discussion*. We review the literature, discuss the possible mechanism of tumour transfer and offer advice aimed at avoiding this complication.

**Key words**: *MFH, bone graft, recurrent tumour*

### Introduction

Autologous bone grafting remains a common method of reconstructing bone defects following the surgical treatment of primary bone tumours. Complications from this procedure are not uncommon.[1] Transplantation of tumour cells from the primary site of tumour to the graft donor site is rare but has been reported in different tissues such as the skin and bones.[2–6] However, the exact mechanism of seeding of the primary tumour to graft donor site is unclear and controversial. It is possible that seeding is a result of poor surgical technique allowing direct transfer of tumours from the primary site to the donor site or it may result from haematogenous transfer.

We report a case of seeding of malignant fibrous histiocytoma from the femur to the iliac crest bone graft donor site, review the literature of this unfortunate complication and discuss the possible mechanism of seeding tumour cells to the graft donor site. We offer advice on how to avoid this complication.

### Case report

A 49-year-old man presented to his local hospital with a 3-month history of pain in the left thigh. There was no swelling and haematological tests were normal. Plain X-rays revealed a lytic lesion of the left distal femur (Fig. 1). A trephine biopsy of the lesion was performed by the local surgeon and was reported as showing no evidence of malignancy, but no definite diagnosis was made.

The lesion was curetted and the defect in the distal femur packed with autologous left iliac crest bone graft supplemented by blocks of bank allograft. A condylar blade plate was applied over the grafted femur. No operative notes were made in the patient's records to determine whether the graft was obtained before the tumour was curetted or if the surgical instruments had been changed.

Histology of the curettings, however, showed a high grade malignant fibrous histiocytoma. The patient was referred to a specialist orthopaedic centre 3 months after the initial presentation and the histological diagnosis was confirmed. Comprehensive tumour staging studies including serum haematological and biological tests, computed tomography (CT) scan of the chest and whole body bone scintigraphy revealed no metastases. The patient was treated with chemotherapy, surgical resection and endoprosthetic replacement of 85% of the distal femur.

Eighteen months after the initial curettage and donor bone graft the patient developed a painful lump under the iliac crest scar at the site of the previous bone graft. Magnetic resonance imaging (MRI) scan and biopsy of this lesion (Fig. 2) showed a high grade malignant fibrous histiocytoma. Further staging studies revealed no other metastasis. Excision of the tumour in continuity with the involved iliac crest was

Correspondence to: Mr A. Abudu, Oncology Service, The Royal Orthopaedic Hospital NHS Trust, Bristol Road South, Northfield, Birmingham B31 2AP, UK. Tel: 0121-685-4150; Fax: 0121-685-4146; E-mail: sarcomas@rohos.demon.co.uk

1357-714X print/1369-1643 online/00/010057-03 © 2000 Taylor & Francis Ltd

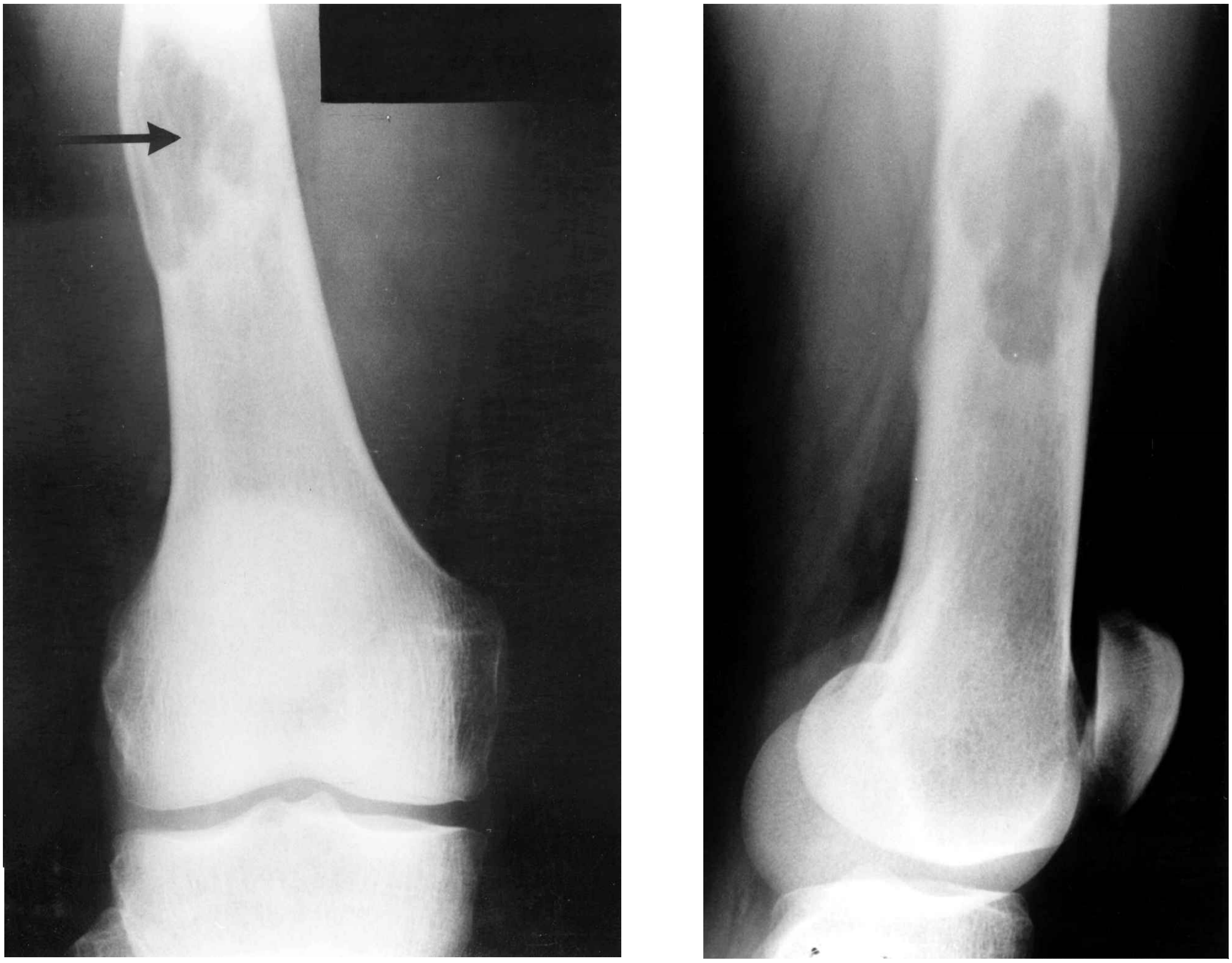

**Fig. 1.** *Plain radiographs of the lesion in the femur (IA antero-posterior view; IB lateral view).*

performed. The patient remained disease free for 3 years, but subsequently developed metastases that led to his death 6 years from the time of initial presentation.

### Discussion

Accurate pathological diagnosis of musculoskeletal tumours is a prerequisite for optimal management of malignant bone and soft tissue tumours. Errors in diagnosis of the biopsy specimen may occur in up to 25% of patients with possible implications on the treatment recommended when biopsy and histological examination are performed at non-oncology centres.[7] Hence, the recommendation that biopsy of suspicious bone and soft tissue lesions and interpretation of histology should be performed at specialist orthopaedic oncology centres with experience in bone and soft tissue sarcoma. This is clearly illustrated by the case presented.

Inadvertent contamination of a wound with tumour cells leads to a risk of local recurrence but the exact incidence of this is unknown. The presence of free tumour cells on surgical instruments and gloves raises the theoretical possibility of direct transplantation of tumour by this route.[8–10] This may represent the mode of metastasis of malignant cells to bone graft donor site when a definitive operation and autologous bone grafting are performed at the same sitting without using different instruments or gloves to obtain the graft.

Experimental evidence exists that trauma may predispose to localization of malignant cells in injured tissues. The exact mechanism of this is unclear and may, at least in part, be due to adherence of tumour cells to damaged endothelium of the micro-circulation at such areas or alteration of blood flow or coagulation mechanism in the traumatized areas.[11–13] Yip *et al.*[6] reported a case of pelvic osteosarcoma with metastases to the fibular donor site 6 years after the initial excision of the pelvic osteosarcoma with pelvic reconstruction using a fibular strut graft at an orthopaedic oncology centre. The fibular graft had been harvested, graft donor site wound closed and instruments changed prior to excision of the pelvic tumour. This case supports the possibility of haematogenous seeding of tumour cells to a surgically traumatized bone graft donor site.

Autologous bone graft donor sites create a suitable tissue surface for eventual transplantation of malignant cells, if bone grafting is performed at the same time as the definitive tumour operation. The

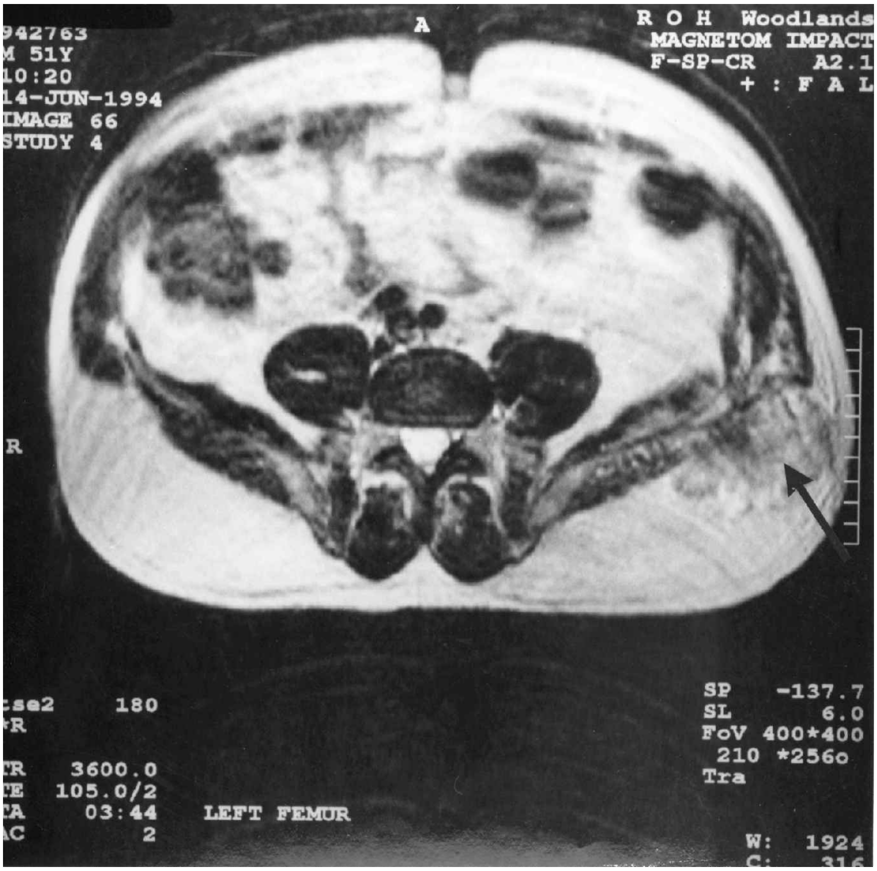

**Fig. 2.** *MRI scan of the pelvis with arrow pointing to the seeded tumour in the iliac wing.*

avoidance of grafting suspicious lesions cannot be overemphasized and where possible the surgeon should avoid autologous graft if alternative means of reconstruction of defeats are available.

There may be situations where autologous bone grafts cannot be avoided. In these situations, meticulous operative technique to minimize seeding tumours is mandatory. These include changing drapes, instruments and gloves when autologous bone graft is harvested following tumour removal, whatever the biopsy report. In our institution we keep the donor site covered with sterile drapes until that part of the case is performed. We change gloves, all instruments, diathermy, suction tips and light handles before bone graft is harvested, where potential seeding of tumour cells could occur. Where possible, the tumour should be excised first and then the graft harvested to prevent a 'congenial soil'[14] being present at the time that potential tumour cells are in the circulation. It is important to document this in the operation record to avoid any future medico-legal implications.

### References

1 Cockin J. Autologous bone grafting-complications at the donor site. *J Bone Joint Surg (Br)* 1971; 53-B:153.
2 Cole GW, Sindelar WF. Iatrogenic transplantation of osteosarcoma. *Southern Med J* 1995; 88:485–8.
3 Kroll SS, Tavollali M, Castello-Sendra J, Pollock RE. Risk of dissemination of cancer to flap donor sites during immediate reconstructive surgery. *Ann Plast Surg* 1994; 33:573–5.
4 Flook D, Horgan K, Taylor BA, Hughes LE. Surgery of malignant melanoma: from which limb should the graft be taken. *Br J Surg* 1986; 73:793–5.
5 Martin-Oliva X, Ballart-Gavila, Fernandez-Suarez M, Navarro-Farre B, Valdes-del-Molino AP. Metastases of an ameloblastoma to the iliac crest. *Int Orthop* 1994; 18:50–2.
6 Yip KMH, Lin J, Kumta SM. A pelvic osterosarcoma with metastases to the donor site of the bone graft. *Int Orthop* 1996;20:389–91.
7 Mankin HJ, Lange TA, Spanier SS. The hazards of biopsy in patients with malignant primary and soft tissue tumours. *J Bone Joint Surg (Am)* 1982: 64A:1121–7.
8 Saphir O. The transfer of tumour cells by the surgical knife. *Surg Gynec Obst* 1954; 63:775.
9 Brandes WW, White WC, Sutton JB. Accidental transplantation of cancer in the operating room. *Surg Gynec Obst* 1946; 82:212.
10 Enneking WF, Maale GE. The effects of inadvertent tumour contamination of wounds during surgical resection of musculoskeletal neoplasms. *Cancer* 1988; 62:1251–6.
11 Alexander JW, Altemeier WA. Susceptibility of injured tissues to haematogenous metastases. An experimental study. *Ann Surg* 1964; 159:933–4.
12 Fisher B, Fisher ER. Trauma and the localisation of tumour cells. *Cancer* 1967; 20:23–9.
13 Agostino D, Eugene EC. Trauma as a cause of localisation of blood borne metastases. Preventive effect of heparin and fibrinolysin. *Ann Surg* 1965; 161:97–101.
14 Paget S. The distribution of secondary growths in cancer of the breast. *The Lancet* 1889; 1:571–3.

## References

[PDF_00189] AGOSTINO D., CLIFFTON E. E. (1965). TRAUMA AS A CAUSE OF LOCALIZATION OF BLOOD-BORNE METASTASES: PREVENTIVE EFFECT OF HEPARIN AND FIBRINOLYSIN.. Ann Surg.

[PDF_00184] ALEXANDER J. W., ALTEMEIER W. A. (1964). SUSCEPTIBILITY OF INJURED TISSUES TO HEMATOGENOUS METASTASES: AN EXPERIMENTAL STUDY.. Ann Surg.

[PDF_00156] Cole G. W., Sindelar W. F. (1995). Iatrogenic transplantation of osteosarcoma.. South Med J.

[PDF_00180] Enneking W. F., Maale G. E. (1988). The effect of inadvertent tumor contamination of wounds during the surgical resection of musculoskeletal neoplasms.. Cancer.

[PDF_00187] Fisher B., Fisher E. R., Feduska N. (1967). Trauma and the localization of tumor cells.. Cancer.

[PDF_00162] Flook D., Horgan K., Taylor B. A., Hughes L. E. (1986). Surgery for malignant melanoma: from which limb should the graft be taken?. Br J Surg.

[PDF_00158] Kroll S. S., Tavollali M., Castello-Sendra J., Pollock R. E. (1994). Risk of dissemination of cancer to flap donor sites during immediate reconstructive surgery.. Ann Plast Surg.

[PDF_00172] Mankin H. J., Lange T. A., Spanier S. S. (1982). The hazards of biopsy in patients with malignant primary bone and soft-tissue tumors.. J Bone Joint Surg Am.

[PDF_00169] Yip K. M., Lin J., Kumta S. M. (1996). A pelvic osteosarcoma with metastasis to the donor site of the bone graft. A case report.. Int Orthop.

